# Economic Segmentation and Health Inequalities in Urban Post-Reform China

**DOI:** 10.3934/publichealth.2016.3.487

**Published:** 2016-08-02

**Authors:** Soyoung Kwon

**Affiliations:** Department of Psychology & Sociology, Texas A & M University, Kingsville, TX, USA

**Keywords:** economic sector, self-rated health, health inequalities

## Abstract

During economic reform, Chinese economic labor markets became segmented by state sector associated with a planned redistributive economy and private sector associated with the market economy. By considering an economic sector as a concrete institutional setting in post-reform China, this paper compares the extent to which socioeconomic status, measured by education and income, is associated with self-rated health between state sector and private sector. The sample is limited to urban Chinese employees between the ages of 18 and 55 who were active in the labor force. By analyzing pooled data from the 1991–2006 *Chinese Health and Nutrition Survey*, I find that there is a stronger association between income and self-rated health in the private sector than in the state sector. This study suggests that sectoral differences between market and redistributive economies are an important key to understanding health inequalities in post-reform urban China.

## 1. Introduction

The association between social status and health, namely social disparities in health, is well established. Specifically, socioeconomically advantaged people exhibit better health and lower rates of mortality than their disadvantaged counterparts [1]. Indeed, Link and Phelan [2] conceptualized socioeconomic status (SES) as a fundamental cause of health disparities because SES embodies “accesses to resources that help individuals avoid diseases and their negative consequences through a variety of mechanisms.” Some cross-national comparison studies further show that strength of the relationship between SES and health varies by institutional contexts [3]. The general finding of the comparison study is that there is a stronger SES-health association in countries with more free–market institutional systems (e.g., United States) and a weaker association in countries with a set of welfare redistribution institutions (e.g., Iceland, Canada, England, Germany) [3],[4]. Extant comparative research typically selects two countries at the polar ends of their chosen typology of either a welfare state regime (liberal, conservative, and social democratic economies) or capitalism (liberal market economies, coordinated market economies, and mixed market economies). A similar distinction exists in transitional China, characterized by sharp differences in economic institutions between the state and private sectors.

Since economic reform was launched in the late 1970s, China has witnessed remarkable economic growth. This economic prosperity improved population health, evident in the decline of mortality rates [5]. However, recent study shows that health gap across social status in China has grown as medical care has deteriorated and income inequality has increased, even as overall public health has been improved [6]. This calls for further academic investigation of health inequalities in reform-era China. Although a large body of market transition literature has focused on the temporal trends in social inequality in various outcomes (e.g., earnings, job mobility) during the reform period, much less attention has been devoted to the sectoral variability of health inequalities.

China provides researchers with a valuable opportunity to examine the role that economic institutions play in shaping health inequalities. During economic reform in China, a fragmented market has emerged, characterized by heterogeneous institutional arrangements and distinctive allocation mechanisms between the state and private sectors [7]–[9]. Considering that the state and private sectors in reform-era China represent both the prototypical planned and market economies, the present study compares health inequalities between the two sectors. An economic institution is key to understanding the patterns of social stratification and inequality. Szelenyi [10] explicitly said that, “…in state socialist societies social inequalities are basically created and structured by redistributive mechanisms.” In capitalist societies, stratification is primarily organized around the market economy [9],[10]. If these two opposing institutions, state socialism and capitalism, coexist in a single society with a form of economic segmentation or dual economic structure, it seems logical to compare the pattern of inequalities between those sectors.

A comparison of the state and private sectors makes it possible to contextualize health inequalities and draw the institutional implications of different economic sectors for health determinations in the reform era. Using data from the Chinese Health and Nutrition Survey, this study investigates the differences in the relationship between SES, measured by income and education, and self-rated health among employees in the private and state sectors in urban China.

## 2. Economic Segmentation in Reform-era China

Economic segmentation theory or dual labor market theory originally evolved to explain socially produced inequality in Western industrial economies and has provided the intellectual underpinning for research on stratification [7]. It has focused on differential returns to human capital (e.g., education, job experiences) between core and periphery sectors. Scholars also demonstrate that the segmentation hypothesis is applied for transitional economies [7],[11]–[14]. Lin and Bian [15], for example, argued that segmentation theory could be directly used to analyze the organizational and labor structures in socialist planned systems because segmentation is “a universal phenomenon associated with all complex societies and all political economies.” China's transitional economy is characterized by “fragmented markets,” the coexistence of a state and private sector [8]. The state sector under the direct supervision of political institutions operates according to the principles of the central planning system, whereas there are more developed market mechanisms in the private sector [16]. Empirical evidence of economic segmentation includes higher earning returns to education in the private sector than in the state sector [7],[16]–[18]. In studying the effect of work organizations on earning inequality in reform-era China, Wu [14] accordingly argues that “earnings may vary significantly among people with the same level education working at the same occupations solely because of their different organizational affiliations.” Empirical observations of sectoral variations in earning determinants are attributable to the economic instructions embedded in the sector.

The extant research, building upon economic segmentation theory, has largely focused on socioeconomic outcomes in the labor market, such as earnings and occupational mobility. However, the segmented labor market structure has received little attention in the health literature. Lobao [19] suggests that a possible extension of the economic segmentation framework to include health outcomes by stating, “there have been virtually no attempts to study the effects of labor market structures on health, education, and other non-economic indicators although this would be a logical research extension that could contribute to a broader understanding of local quality of life.” Only a few studies have been undertaken to compare noneconomic outcomes between two sectors, revealing that Chinese employees in the state sector tend to feel happier than employees in the private sector [18],[20]. Sectoral difference in happiness is explained by sharp differences in fringe benefits and job security between sectors. The present study moves beyond direct comparison of outcomes between sectors that focused on sectoral inequality and examines how the pattern of health inequalities differs by sectors with distinctive institutional settings by comparing the association of education and income with health. In other words, the present study focuses on institutional dissimilarities, in the way health status for workers in the state and private sectors are rewarded differently, and draw on the institutional implication for social stratification and inequalities in reform-era urban China.

## 3. Economic Segmentation and Health Inequalities

Among the many possible indicators of socioeconomic status, this study focuses on the effect of education and individual income on self-rated health. In medical sociology, the health advantages associated with educational attainment have been an analytic focus in understanding the mechanisms and patterns of health inequalities. It has been well documented that higher education is positively associated with better health [1],[21]–[23]. However, educational attainment does not lead to the same degree of health advantage across societies because institutional arrangements exacerbate or alleviate socioeconomic inequality and the extent to which socioeconomic inequality affects the procurement of essential resources for health [4],[22].

In light of comparative social stratification perspectives, the economic rewards of an education tend to be higher in an industrialized market economy than in the state-socialist redistributive economy, illustrating distinctive institutional logic embedded in economic institution [8],[9]. Because economic reforms have introduced a market mechanism into Chinese society, the market transition literature has focused on economic returns from education, which allows for assessing change in the mechanism of resource allocation during market transition in China [8],[24]. Advocates of market transition theory propose that the introduction and expansion of a market economy mechanism that favors efficiency and productivity would result in increasing returns from education [24]. The outcome variable of interest is health status; thus it is important to consider both tangible (i.e., employment and income) and intangible (i.e., health behavior, knowledge, and self-efficacy) gains associated with education [22]. Intangible resources accrued from education may not be necessarily different across sectors. However, when contextualizing the interrelationship between education, economic conditions, and health within the context of the Chinese segmented economy, the private sector is expected to exhibit a more salient association between education and health than the state sector.

Comparative study on the effects of income indicates that redistribution policies in Iceland ameliorate social stratification and inequality; thus, relative affluence does not provide extra health benefits for wealthy Icelanders [3]. In the liberal market economy (e.g., the United States), the distribution of resources is left to the market, which produces more prominent income-based inequality in health. The reason for the strong association between income and health in a liberal market economy is that “individuals with income advantage in a higher-income inequality society may have more resources that they can translate more effectively into better health, and the poor would be more deeply disadvantaged”[25].

Within an economic sector, the distribution of income is closely tied to reward structures. Chinese employees in the state sector where egalitarian socialism and a redistributive economy prevail are generally provided with extensive welfare benefits and a fairly equal wage distribution regardless of the work organization's profitability or individual productivity, although there is hierarchical rank [26]. Workers in the state sector in urban China enjoy significantly more fringe benefits than their counterparts in the private sector [14]. Unskilled workers in state-owned enterprises have higher earnings compared to unskilled workers in the private sector. In contrast, a skilled worker in the state sector earns less than those in the private sector [7]. Further, the state work organization commonly engages in handing out monetary benefits to their workers [27]. In income distribution, private enterprises were at both the highest and lowest ends, indicating great variation within the private sector [28]. On the other hand, income in the private sector is largely determined by performance at work, thus resulting in large variations of wages among employees. This pattern of within-sector income distribution is expected to influence the strength of the relationship between income and health — where a stronger effect of income on health exists in the private sector compared to the state sector.

## 4. Materials and Methods

### 4.1. Data

The data to be analyzed are from the *China Health and Nutrition Survey* (CHNS), an unbalanced panel data. It is a multistage random cluster sampling from 9 provinces (Guangxi, Guizhou, Heilongjiang, Henan, Hubei, Hunan, Jiangsu, Liaoning, and Shandong) that vary substantially in socio-economic development and public resources. The main goal of the survey is to scrutinize how the social and economic transformation of Chinese society affects the health and nutritional status of its population. In addition, the survey also provides rich information about household members aged 18 and older, including employment details suitable for the current research focus.

Beginning in 1989, six additional waves were collected in 1991, 1993, 1997, 2000, 2004, and 2006. The present study did not include the first wave, the 1989 survey, due to the absence of a self-rated health variable. To focus on the central thesis of sector variations and health disparities, I pooled across six waves and used all available data from members of sampled households who are between ages 18 and 55 and active in the labor force in urban China. I have selected age 55 as a cut point to avoid issues of retirement, which typically occurs around this time, thus proactively avoiding a change in activity level due to this life change. Furthermore, inclusion of respondents over retirement age and currently working may result in a health selection bias. Only respondents with complete information on all variables were included in the multivariate analyses, and the final sample size was 5,091.

### 4.2. Measures

#### 4.2.1. Health

Self-rated health status was measured by the question: “Right now, how would you describe your health compared to that of other people your age?” Respondents selected one of four answers: “1 excellent, 2 good, 3 fair, 4 poor”. These answers were reverse coded. Self-rated health has been considered a good indicator of objective health [29], and its validity has been tested in the Chinese population [30].

#### 4.2.2. Economic Sector

In the CHNS dataset, respondents were asked to classify the “type of work unit” with regards to their primary job. Economic sector is distinguished by ownership of the work unit. The state sector includes state enterprises and institutions and large collective enterprises owned by county, city, and province. The private sector includes enterprises and institutions that individuals own, foreign investors or other private parties. In short, to capture differences in the pattern of health disparities between the market and redistributive economies, work sector was coded as a dummy variable, state sector versus private sector.

#### 4.2.3. Socioeconomic Status

As a proxy of social status, both education and individual income were included in this study. *Education* was measured by four categories corresponding to the level of education attained (1: primary school or lower; 2: junior high school; 3: senior high school or higher). The primary school or lower educational level serves as reference category. *Individual income* includes the sum of all income sources and is categorized by quartiles.

#### 4.2.4. Controls

I also include control variables and other covariates that may affect health status and be correlated with sector: age, sex, marital status, occupation, *hukou* status (household registration system), geographical locations, and time. *Gender* was coded 0 for males and 1 for females. *Marital status* was treated as a dichotomous variable, married versus not. I identified occupations as managers/administrators/executive, professionals and office workers while utilizing other manual/ordinary workers as a reference group. Because of regional variations in socioeconomic conditions and the pace of market economic reform [31], geographic region was controlled. It was categorized into a coastal region, northeastern region, inland region, and mountainous south region.

Finally, as health-risk behaviors, *smoking* and *alcohol consumption* were also included. The smoking variable was categorized into current smokers versus non-smokers; and alcohol consumption was also coded as a binary variable indicating whether the respondent consumed any alcohol during the last year. The analysis controls for time since the data for multiple waves were pooled for the analysis. Table 1 reports descriptive statistics of covariates in each sector.

### 4.3. Analysis

This study uses repeated ordinal categorical response data in a longitudinal study. Accordingly, it performed a logistic random intercept, multilevel model with measurement occasions (level-1) that were nested within individuals (level-2). The model allows accounting for temporally hierarchical structures, thus assessing the effect of covariates at different levels on the outcome variable. Further, a multilevel model corrects for biases in parameter estimates and standard errors because it accounts for clustered data with residual components at each level.

The plan for the analysis is as follows: (a) estimate mixed ordinal logistic regression of self-rated health on education and interaction term between education and sector, net of age, gender, marital status, and time (Model 1); (b) estimate mixed ordinal logistic regression of self-rated health on income and interaction between income and sector, net of age, gender, marital status, and time (Model 2); and (c) estimate mixed ordinal logistic regression of self-rated health on income, education, its interaction terms with sector, and net of all other covariates (Model 3). It should be noted that income and education are entered in the model separately because inclusion of income could absorbs part of the effect of education, vice versa [32],[33]. All statistical analyses were conducted using Stata 14.0 [34].

## 5. Results

Table 1 displays the descriptive statistics of the study sample; currently employed Chinese adults aged 18–60. On average, respondents who reported their health status as “excellent/good” was relatively high, 76.84 percent. Using pooled data, 67.72 percent of the respondents were employed in the state sector; however, across periods, the percentage of state sector employees declined from 80.60 percent in 1991 to 59.09 percent in 2006. This decline reflects the growing employment rate in the private sector as market economic reforms have increased over time (not shown). Health status showed no significant differences in self-rated health by sectors. This may reflect the pros and cons of working in the state and private sector, which possibly offsets any difference in health status between state and private sector employees. Specifically, factors relevant to positive health include generous benefits and a stable work environment, but low wages in the state sector may be relevant to negative health outcomes. This is partially evident in the income descriptive statistics, showing that employees in the private sector generally enjoy higher income, but there is more heterogeneity in income within the private sector (SD = 15,162 in the private sector; SD = 11,291 in the state sector). Meanwhile, workers with the highest educational attainment (i.e., ≥ senior high school) were concentrated in the state sector, which may indicate a preference for an employment in the state sector.

Table 2 reports the estimated association of income and education with self-rated health and its interactive effects with economic sector among urban Chinese employees. As shown in Model 1, a negative coefficient (or less than 1 odds ratio) for the interaction between education and sector shows that the effect of education is less prominent in the state sector than in the private sector. However, this interaction is statistically non-significant.

**Figure 1.**
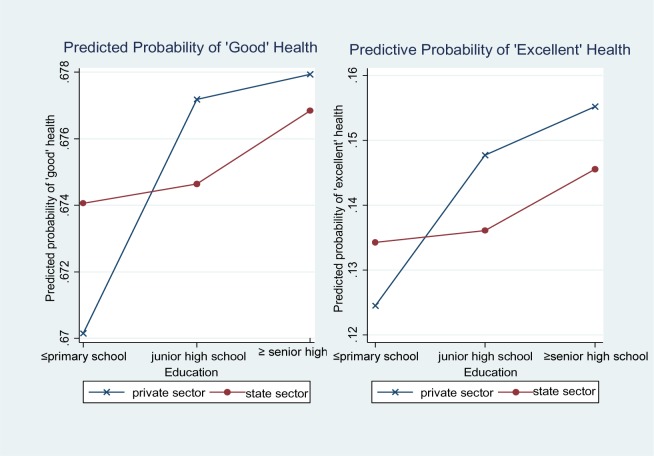
Predicted probabilities of good and excellent self-rated health by education.

Note that all interaction term results reported in Table 2 were primarily tested in terms of a multiplicative scale [35]. Based on Model 1, I calculated the probability of good and excellent self-rated health by using marginal effect at the means. As displayed in Figure 1, the slope for the private sector is much steeper than for the state sector, indicating greater health differences by education in the private sector than in the state sector.

**Figure 2.**
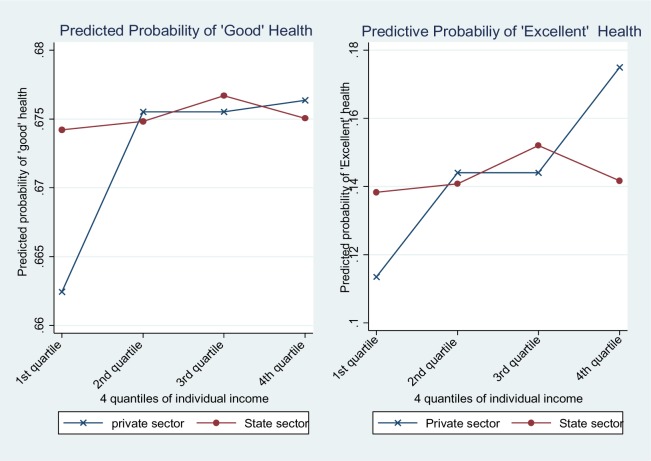
Predicted probabilities of good and excellent self-rated health by income.

The income-based differential in health showed that a higher income level is positively associated with better self-rated health, and its positive effect is much less prominent in the state sector (OR = 0.614 C.I 0.392–0.899) (see Model 2). Based on Model 2, I calculated the probability of good health and excellent health (see Figure 2). As shown in Figure 2, the slope for the private sector is much steeper than for the state sector. Along with the statistical significant interaction term, this figure clearly demonstrates stronger effect of income on health in the private sector than in the state sector. Finally, Model 3 includes income, education, and their interactive terms together with all other covariates. Inclusion of all those variables did not alter the main findings from Model 1 and Model 2.

**Table 1.** Means/Percent and S.D for All Variables, Pooled Data from CHNS, 1991–2006 (N = 5,091).

Variables		Total (*N* = 5,091)	State Sector (*N* = 3,538)	Private Sector (*N* = 1,553)
Self-rated Health status (% good/excellent)		76.84	76.71	78.03
State sector (%)		67.72		
*Education (%)*				
≤ primary school		9.45	5.51	18.42***
Junior high school		29.62	23.37	43.85***
≥ Senior high school		60.93	71.11	37.73***
*Individual Income (quartile income)*				
1^st^ quartile		17.95	18.09	17.64
2^nd^ quartile		22.25	20.80	22.89
3^rd^ quartile		29.09	28.46	30.52
4^th^ quartile		30.70	30.55	31.04
*Individual income ^a^*		9593.6	9161.1	10579.3***
		(12614.4)	(11291.7)	(15162.3)
*Occupation* (%)				
Manager		11.33	14.76	3.49***
Professional		22.47	30.65	3.81***
Staff		14.44	19.01	4.01***
Household registration status (*Hukou*)		94.52	98.16	86.22***
Insurance status		58.30	75.64	18.80***
*Demographic characteristics*				
Age(years, mean)		37.49	38.68	37.32
		(10.14)	(8.90)	(10.21)
Gender (Male %)		42.64	42.25	43.12
Marital Status (married, %)		81.55	81.48	81.64
*Residential location* (%)				
Coastal region		18.30	15.74	24.14
Northeastern region		22.72	28.41	9.78
Inland		32.74	34.05	29.74
Mountainous south region		26.22	21.79	36.31
*Health-risk behaviors*				
Smoking (current smoker%)		34.00	34.43	33.03
Alcohol consumption (drink alcohol in previous year)		44.37	47.34	37.60 ***

Note: ^a^ continuous income level was only reported in the descriptive statistics while quartile income level was included in the regression analyses. Standard deviations in parentheses.

*** *p* < 0.001 (two-tailed test and chi-square test).

**Table 2.** Ordinal ogistic Mixed Regression of Self-Rated health on Education and Income by Sector, CNHS 1991–2006 (N = 5,091).

				Model 1			Model 2				Model 3						
				OR	95% C.I		OR		95% C.I		OR			95% C.I			
*Fixed effects*																	
State sector				1.092	0.765–1.558		1.260†		0.959–1.654		1.377			0.824–2.299			
Education																	
Junior high school				1.223	0.901–1.652						1.020			0.731–1.423			
≥ Senior high school				1.298	0.948–1.178						1.035			0.729–1.468			
Individual income																	
2^nd^ quartile income							1.322†		0.960–1.819		1.255			0.875–1.801			
3^rd^ quartile income							1.322†		0.979–1.785		1.192			0.852–1.669			
4^th^ quartile income							1.675***		1.229–2.282		1.499*			1.062–2.117			
*Interactions*																	
Sector × Junior high educ.				0.830	0.549–1.255						0.811			0.497–1.324			
Sector × ≥ Senior high educ.				0.847	0.563–1.275						0.931			0.572–1.516			
Sector × ≥ 2^nd^ quartile							0.772		0.536–1.113		0.864			0.562–1.329			
Sector × ≥ 3^rd^ quartile							0.846		0.594–1.204		0.907			0.605–1.359			
Sector × ≥ 4^th^ quartile							0.614**		0.424–0.888		0.594*			0.392–0.899			
*Random effect*				1.112***			1.124***				0.941***						
Log-likelihood				–6603.7333			–6627.874				–5017.881						

*Note*: Model 1 and 2 controlled for age, gender, marital status and survey year. Model 3 as a full model further controlled for region, health behavior, occupation, *hukou* status, and insurance status. C.I = Confidence Interval.

† *p* < 0.10; * *p* < 0.05; ** *p* < 0.01; *** *p* < 0.001 (two-tailed test).

## 6. Discussion and Conclusions

Economic segmentation theory has been utilized largely to analyze inequalities in socioeconomic outcomes; however, little attention has been paid to the possibility that location within the segmented economy matters when studying health disparities. By recognizing dual economic structure segmented principally by state and private sector in reform-era China, this study adopts sectoral approach to explore the institutional implications of different economic sectors for health determinants.

The analysis reveals that health status associated with education is not statistically different between sectors. Meanwhile, differences in the marginal effects of education between sectors showed that economic sectors modified the relationship between education and health in urban China. Separate, supplementary analyses for the employees only in the state sector and private sector were also performed, showing significant association of education with health in the private sector, though not in the state sector (not shown). Taken together, the result does not provide strong evidence for the sectoral variations of education-health association; however, it does provide unique insights into the pattern of health inequalities in each sector.

In light of market transition theory, the varied association is assumed to be in part due to the economic institutions embedded in each sector. For example, human capital (i.e., educational attainment) tends to be highly rewarded in a market economy [8],[11]. Market transition theory, which was originally developed and extensively applied to study earning inequalities, tends to focus on the material pathway through which education is associated with health in the private sector. However, the health advantage associated with education is not solely explained by the income [22],[33]. Considering that education represents the acquisition and interpretation of health information, it is possible that health-promoting behaviors and healthy choices would provide additional health benefits in the private sector because health services for employees in the private sector are not as comprehensive are those for those in the state sector. Another finding is that health differential by income is more pronounced in the private.

This study finds statistically significant differences in the association between income and health between sectors. Income has a weaker association with health in the state sectors than in the private sectors. This sectoral gap presumably reflects redistributive mechanisms in the state sector but a competitive market mechanism in the private sector. The finding seems to be quite consistent with cross-national variations in health inequalities. A cross-national literature shows that the strength association varies across institutional contexts and dimensions of social stratification [4],[36]. Specifically, cross-national variation in the social organization of health care and in the welfare state exerts a buffering effect on the association between income and health [4]. In a similar vein, comprehensive fringe benefits, including medical care, in the state sector might serve as mechanisms to ameliorate income-based health differentials. Because previous research has largely focused on income determinants, little is known about how income operates as a health determinant differently across economic sectors. A sectoral approach that focuses on the impact of economic sectors on social stratification order contributes to our understanding of differential health returns according to income in the state sector and private sector.

Several limitations exist within the study. First, I did not specify what characteristics of the economic sector might explain variations in the patterns of health disparities. Assuming that an economic sector based on ownership in a Chinese context represents a distinctive institutional setting, the present study employed conceptual multilevel analyses to compare the patterns of health disparities between sectors. Future research could incorporate organizational-level characteristics to examine their implications for the health disparities. These characteristics can become differentiated and complicated in various ways, in terms of profitability among work organizations, industry sectors, and even local labor markets. Similarly, the present study did not identify the mechanism underlying differential linkages between SES and health by sectors. Another limitation concerns the absence of information on the specific work organization's history, and thus I did not control those in a privatized state work organization. Finally, although the inverse relationship between SES and health has been well documented, the nature of the causal relationship has not been clearly elucidated. Specifically, there are two competing hypotheses for the SES-health relationship: the “social causation” hypothesis versus the “health selection” hypothesis [37]. The health selection hypothesis stipulates that individuals' health affects their chance to attain upward SES mobility. Health selection cannot be ruled out because it can serve as a driver of health inequalities. Health differentials among workers in the private sector may contribute to unequal income distribution within the sector. However, I believe there is little evidence on the health selection, that is, what aspects of the economic sector or work organization strengthen or weaken the health benefits that accrue to income and/or education.

By estimating and comparing the association of income and education with health across redistributive and market economies of the state and private sectors, this study provides a glimpse into how specific institutional context shapes the extent to which resources and social standing influence self-rated health among employees in urban China. This study also demonstrates the applicability of economic segmentation theory to the sectoral variations in health disparities. By this logic, it is quite possible that the strength of the relationship between income and health will increase in the future if the current economic structure undergoes changes, such as with the introduction of a market economy mechanism into the state sector or further reform of state-owned enterprises.
